# Auditory and visual distractors disrupt multisensory temporal acuity in the crossmodal temporal order judgment task

**DOI:** 10.1371/journal.pone.0179564

**Published:** 2017-07-19

**Authors:** Cassandra L. Dean, Brady A. Eggleston, Kyla David Gibney, Enimielen Aligbe, Marissa Blackwell, Leslie Dowell Kwakye

**Affiliations:** Department of Neuroscience, Oberlin College, Oberlin, Ohio, United States of America; Emory University, UNITED STATES

## Abstract

The ability to synthesize information across multiple senses is known as multisensory integration and is essential to our understanding of the world around us. Sensory stimuli that occur close in time are likely to be integrated, and the accuracy of this integration is dependent on our ability to precisely discriminate the relative timing of unisensory stimuli (crossmodal temporal acuity). Previous research has shown that multisensory integration is modulated by both bottom-up stimulus features, such as the temporal structure of unisensory stimuli, and top-down processes such as attention. However, it is currently uncertain how attention alters crossmodal temporal acuity. The present study investigated whether increasing attentional load would decrease crossmodal temporal acuity by utilizing a dual-task paradigm. In this study, participants were asked to judge the temporal order of a flash and beep presented at various temporal offsets (crossmodal temporal order judgment (CTOJ) task) while also directing their attention to a secondary distractor task in which they detected a target stimulus within a stream visual or auditory distractors. We found decreased performance on the CTOJ task as well as increases in both the positive and negative just noticeable difference with increasing load for both the auditory and visual distractor tasks. This strongly suggests that attention promotes greater crossmodal temporal acuity and that reducing the attentional capacity to process multisensory stimuli results in detriments to multisensory temporal processing. Our study is the first to demonstrate changes in multisensory temporal processing with decreased attentional capacity using a dual task paradigm and has strong implications for developmental disorders such as autism spectrum disorders and developmental dyslexia which are associated with alterations in both multisensory temporal processing and attention.

## Introduction

### Temporal influences on multisensory integration

As we interact with the world around us, we encounter many stimuli that are perceptible to multiple senses. The field of multisensory integration studies the neurological processes that combine these disparate unisensory stimuli into one unified perception of the world and the resulting changes in perception and behavior [1]. Several stimulus features modulate the likelihood and strength of multisensory integration and have been termed the principles of multisensory integration. For example, unisensory stimuli that share a close temporal and spatial correspondence are more likely to be integrated [2,3]. Additionally, greater integration has been observed in response to stimuli that are relatively less salient [4]. Evidence for the importance of the temporal principle was first established in multisensory neurons in the superior colliculus (SC) of anesthetized cats [3]. Two unimodal stimuli presented closely in time were more likely to produce a response that was superadditive relative to the sum of both unisensory components [5]. Furthermore, the magnitude of the multisensory enhancement decreased as the paired stimuli are presented at larger temporal asynchronies, although some neurons respond most strongly to particular temporal offsets between unisensory stimuli [3]. This effect has been demonstrated for audiovisual, visual-somatosensory, and auditory-somatosensory stimulus pairs [4]. The temporal principle has also been shown to apply to human perception, and several constructs have been developed to quantify differences in multisensory temporal processing [6,7]. The temporal window of integration describes the interval of time over which two stimuli may be perceptually bound into a unified percept, and this window has been shown to differ across individuals [8], recalibrate based on task demands [9–11], and narrow due to training [6,12–14]. Closely related to the temporal window of integration is the concept of crossmodal temporal acuity which describes the amount of time necessary for a participant to distinguish temporal features across sensory modalities [8,15]. Importantly, disruptions in the temporal processing of multisensory information have been strongly linked to several developmental disorders including autism spectrum disorder, dyslexia, and schizophrenia [16–19]. Multisensory temporal processing is also known to develop across childhood and reach adult-like levels in adolescence [20,21].

### Top-down and attentional influences on multisensory integration

In addition to the bottom-up stimulus features discussed in the previous section, several top-down processes such as attention also interact with and modulate multisensory integration (for general review see [22]). In crossmodal attentional cuing, a stimulus in one sensory modality can spatially direct attention to benefit the processing of a target in a different modality [23–26]. Similarly, attentional resources that are captured by a stimulus in one modality can spread to an unattended stimulus in another modality as long as they share a high temporal correspondence [27–30]. Lastly, a non-spatial, task irrelevant auditory or tactile stimulus can direct attention to a visual target in a complex, dynamic environment [31,32].

Several studies have also investigated whether multisensory integration can occur pre-attentively or is dependent on top-down attentional processes. While some studies suggest that attention is necessary for the integration of multisensory stimuli [33–38], other studies provide evidence that integration is independent of the effects of attention [39–42]. Aspects of the multisensory stimulus may modulate whether attention is necessary for multisensory integration. For example, multisensory speech integration has been consistently shown to lessen under high attentional demands [36–38]; however, emotional multisensory stimuli may be integrated pre-attentively [41]. Additionally, multisensory stimuli of varying modalities are more effective at capturing exogenous attention, particularly in highly distracting circumstances [43,44]. However, a recently published study has shown that attention is necessary for multisensory integration regardless of the complexity of the multisensory information being integrated [38].

### Interaction between multisensory attention and temporal processing

As discussed above, both bottom-up features, such as the temporal relationship between unisensory stimuli, and top-down processes such as attention influence the likelihood that unisensory stimuli will be perceptually combined. A growing number of studies have begun to explore how multisensory temporal processing and attention interact to inform our understanding of multisensory events in our environment. A group of studies have found that the crossmodal effects of attention decrease with increasing temporal disparity between the unisensory subcomponents [30,31,45]. For example, the crossmodal spread of attention between an attended stimulus of one modality to an unattended stimulus of another modality decreases as the two stimuli are separated in time [30].

Attention also alters the speed of processing of stimuli such that attended objects come to our conscious awareness earlier than unattended objects. This phenomenon is described by the law of prior entry [46]. In a multisensory context, when attention is directed to a single modality, objects in that modality will be perceived earlier than objects in another modality. This prior entry effect has been observed across several modality pairings [47–52]. Prior entry in a crossmodal context is usually assessed using crossmodal temporal order judgment (CTOJ) or simultaneity judgment (SJ) tasks. In these tasks, participants either judge the temporal order (CTOJ) or simultaneity (SJ) of stimuli across two modalities that are separated by varied stimulus onset asynchronies (SOA). For both CTOJ and SJ tasks, a point of subjective simultaneity (PSS) can be determined that represents the temporal relationship between the two unimodal stimuli that is perceived as simultaneous by the participant. If a participant is directed to specifically attend to one modality, the PSS will shift toward the participant perceiving the attended modality earlier [46].

Multisensory researchers have begun to explore how attention may alter multisensory temporal processing by changing the temporal window of integration or crossmodal temporal acuity. A previous study conducted by Vatakis and Spence (2006) presented paired visual and auditory stimuli at various SOAs within a stream of unimodal or multimodal distractors to investigate temporal crowding in a CTOJ experiment. They observed changes in crossmodal temporal acuity (increases in the just noticeable difference (JND)) as a function of position in the distractor stream and the modality of the distractor stream with audiovisual distractors disrupting TOJ performance the most. The results of this study demonstrate that temporal crowding may decrease crossmodal temporal acuity [53]. Alternatively, Van der Burg et al investigated the effects of spatial crowding on crossmodal temporal acuity in a novel synchrony judgment task. Participants viewed complex and dynamic stimuli, 19 discs uniquely modulating in luminance one of which matched an amplitude modulated tone, while judging which visual stimulus was synchronous to the tone. Synchrony judgment performance was unchanged by number of discs indicating that visual spatial crowding does not significantly alter crossmodal temporal acuity [54]. Donohue et al sought to determine whether attention would influence the size of the temporal window of integration. They used a selective attention paradigm for which attention was directed to the left or right hemisphere, and stimuli could be attended or unattended (i.e. occurring in the attended or unattended hemisphere). Three distinct behavioral tasks gave three different patterns of interactions between attention and the temporal window of integration, indicating that the effect of attention on multisensory temporal processing is complex [55].

### Current study questions and hypotheses

Although a handful of studies have investigated the links between attention and multisensory temporal processing, their lack of consistency suggests that we are far from a complete understanding. Thus far, no studies have investigated changes in crossmodal temporal acuity while increasing attentional load. Similar dual-task study designs have revealed that an attentionally demanding secondary task can decrease multisensory integration [37,38,56]. Additionally, only one study has investigated whether distractor modality differentially impacts multisensory temporal processing [53]. The present study investigated whether increasing attentional load would decrease crossmodal temporal acuity in a CTOJ task by utilizing a dual-task paradigm. Participants were asked to judge the temporal order of a flash and a beep presented at various SOAs while also directing their attention to a secondary distractor task, in which the subject must detect a target stimulus within a stream of visual or auditory distractors. We hypothesized that crossmodal temporal acuity would decrease with increasing load and that the modality of the distractor would modulate the extent of the effect for visual—leading versus auditory-leading stimulus pairs. We did find decreases in crossmodal temporal acuity with increasing attentional load; however, these effects were indistinguishable across distractor modalities.

## Materials and methods

### Participants

A total of 88 (55 females, 18–38 years of age, mean age of 22) typically developing adults are included in the data analysis for this study. 73 (44 females, 18–38 years of age, mean age of 22) participants completed the CTOJ task along with visual distractors (RSVP experiment), and 29 (17 females, 18–28 years of age, mean age of 21.5) completed the CTOJ task along with auditory distractors (RSAP experiment). 14 participants completed both experiments in separate sessions. Some participants completed additional experimental tasks while completing the current study procedures. Participants were excluded from final analysis if they did not complete all load conditions for either the RSVP or RSAP experiment [RSVP: 9 participants (7 females, mean age of 20.0); RSAP: 0 participants] or did not have a total accuracy of at least 70% on the distractor task for both load conditions [RSVP: 4 participants (3 females, mean age of 20.8); RSAP: 19 participants (14 females, mean age of 21.1)]. Participants reported normal to corrected-to-normal hearing and vision and no history of developmental disorders or seizures. Participants gave written informed consent and were compensated for their time. Study procedures were approved by the Oberlin College Institutional Review Board and were conducted under the guidelines of Helsinki. Data was collected for the RSVP experiment from June 2013 through July 2014 and for the RSAP experiment June 2014 through January 2015. Participants were recruited through flyers distributed across and the Oberlin College campus and online for the Oberlin community. Potential participants contacted the lab through email or phone to receive more information about study participation and to schedule an appointment if interested in participating.

### Experimental design overview

All study procedures were completed in a dimly lit, sound-attenuated room. Participants were monitored via closed-circuit cameras for safety and to ensure on-task behavior. All visual stimuli were presented on a 24” Asus VG 248 LCD monitor at a screen resolution of 1920 x 1080 and a refresh rate of 144Hz that was set at a viewing distance of 50cm from the participant. All auditory stimuli were presented from Dual LU43PB speakers which were powered by a Lepai LP-2020A+ 2-Ch digital amplifier and were located to the right and left of the participant. Stimulus and SOA durations were confirmed prior to data collection using an oscilloscope and photodiode to measure visual stimuli. SuperLab 4.5 software was used for stimulus presentation and participant response collection. Participants indicated their responses on a Cedrus RB-834 response box, and responses were saved to a text file.

This study employed a dual task design to determine whether distracting attention from a multisensory task would alter crossmodal temporal acuity and whether this effect depended on the modality of the distractor. Similar dual task designs have been shown to reduce attentional capacity [57–59]. Participants completed a primary crossmodal temporal order judgment (CTOJ) task and were also presented with either a rapid serial visual presentation (RSVP) stream or a rapid serial auditory presentation (RSAP) stream in three conditions of increasing perceptual load. Participants were asked to detect a target stimulus within the RSVP or RSAP stream while they completed the CTOJ task. Perceptual load was varied for the distractor tasks to titrate the attentional resources distracted from the CTOJ task. All study procedures related to each distractor modality were completed together. Participants completed the CTOJ task at varying perceptual loads of the distractor task, and each load condition was separated into blocks. Further, the order of the load condition blocks was randomized across participants. Thus, each block tested a particular distractor modality by perceptual load condition. For each block, participants first practiced the CTOJ task without any distracting stimuli. They then practiced the CTOJ task with the additional instructions for that perceptual load.

### Crossmodal temporal order judgment task (Fig 1A)

**Fig 1:**
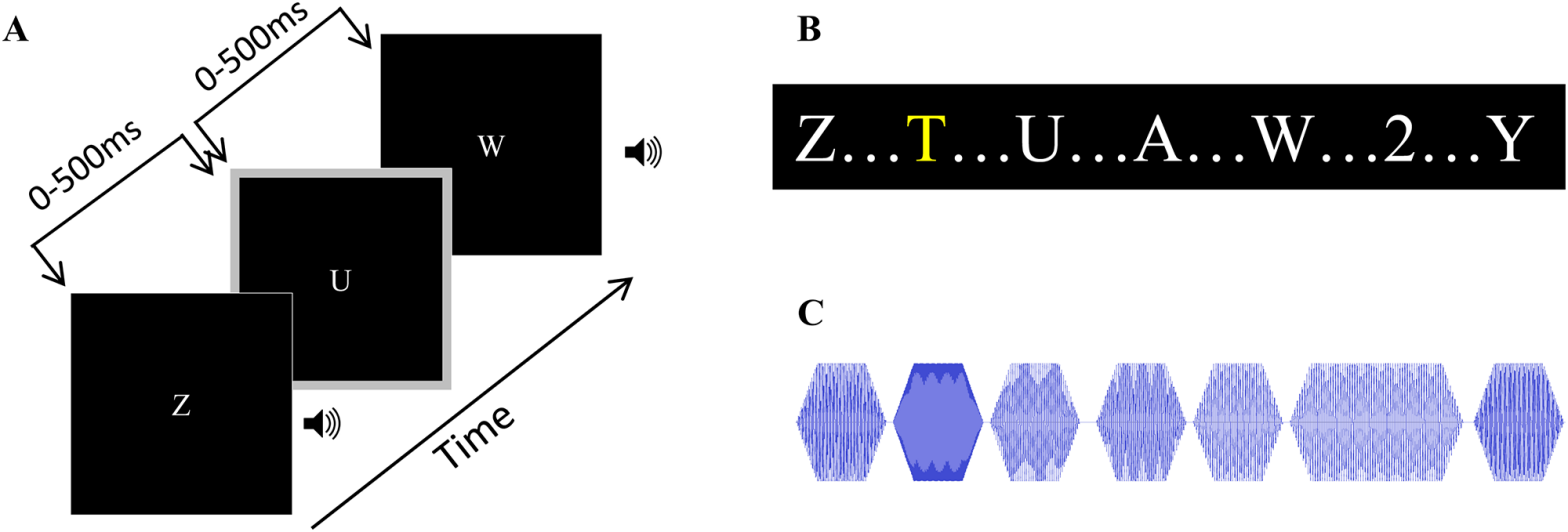
Experimental design and stimuli. A: Participants completed a CTOJ task during which they determined whether a flash (gray border at the edge of the screen) or a beep occurred first. SOAs ranged from -500–500 with negative SOAs indicating that the beep occurred first. B: Some participants completed the CTOJ task while completing a secondary task with visual distractors. Participants were instructed to either ignore the distractors (NL), report a yellow letter (LL), or report a number (HL). C: The remaining participants completed the CTOJ task while completing a secondary task with auditory distractors. Participants were instructed to either ignore the distractors (NL), report a tone that was two octaves above the standard tones (LL), or report a tone that was twice the length of the standard tone (HL).

Visual stimuli consisted of a gray flash at the border of the screen subtending 1.7° from the edge of the screen. (Fig 1A) The flash was presented 28.1° horizontally and 15.9° vertically from central fixation for 21ms. Auditory stimuli consisted of a 3500Hz pure tone beep presented centrally for 21ms at 70dB SPL. For each trial, there was a 500ms pre-stimulus interval during which either an RSVP or RSAP stream was presented. For negative SOA trials, the beep was then presented followed by the flash at varying SOAs. For positive SOA trials, the flash was presented before the beep at varying SOAs. The SOA increments were: -500, -400, -300, -200, -150, -100, -50, 0, 50, 100, 150, 200, 300, 400, and 500 ms. The SOA of 0ms indicates that the auditory and visual stimuli were presented simultaneously. Positive and negative SOA trials were repeated eight times per block across two blocks for a total of 16 trials. Simultaneous trials were repeated 16 times per block across two blocks for a total of 32 trials. The RSVP or RSAP stream continued during the presentation of the CTOJ stimuli and for 500ms after. Then, a response screen was presented that asked “which came first?” Participants indicated their response with a “flash” or “beep” button press. Once participants responded to the CTOJ task, they were asked to report with a “yes” or “no” button press whether they detected a target in the RSVP or RSAP streams in the LL and HL blocks. In the NL block, the next trial started after the participant reported on the CTOJ task. Participants first completed a practice round to establish baseline accuracy for each block. In the practice round, each trial was repeated until participants could correctly identify whether the flash or beep came first. The practice round included -500, -400, -300, 300, 400, and 500 ms SOAs. After completing the practice, participants completed two identical blocks and were given the opportunity to take a short break between blocks. The trials within blocks were presented in random order.

### Visual distractor task (Fig 1B)

This visual distractor task was similar to the previously reported methods in Gibney et al 2017 [38]. (Fig 1B) Stimuli consisted of rapid serial visual presentations (RSVP) of white and yellow letters and white numbers subtending a 3.5° visual angle and presented at center. Some letters (I, B, O) and numbers (1, 8, 0) did not appear in the RSVP streams because the visual similarity between the letters and numbers would be confusing for participants. The RSVP stream was presented continuously before and after the CTOJ stimuli. Each letter/number in the RSVP stream was presented for 100ms with 20ms between letters/numbers. The distractor task included three condition types: no perceptual load (NL), low perceptual load (LL), and high perceptual load (HL). The participant was presented with an RSVP stream and either asked to ignore it (NL), detect infrequent yellow letters (LL), or detect infrequent white numbers (HL). Previously published dual task studies have utilized similar RSVP streams composed of letters and numbers with a color change representing a low load target and/or a number representing a high load target because a color difference is easier to detect than a graphemic difference and would thus require less attentional resources to process [60–63]. Each RSVP stream had a 25% probability of containing no numbers or yellow letters, a yellow letter only, a number only, or a yellow letter and number resulting in a 50% probability of a target being present for the LL and HL conditions. After each trial, participants were asked to respond first to the CTOJ task then report with a “yes” or “no” button press whether they observed a target for that trial. Each load condition was completed in a separate block, and participants were able to take breaks between blocks. The order that participants completed the load condition blocks was randomized and counterbalanced across participants.

### Auditory distractor task (Fig 1C)

Stimuli consisted of rapid serial auditory presentations (RSAP) of musical notes presented centrally at 60dB SPL. (Fig 1C) The musical notes were pure tones whose frequency fell on an accepted musical note in a twelve point scale within the C4-C5 octave (262–523 Hz) range. The RSAP stream was presented continuously before and after the CTOJ stimuli. Each musical note in the RSAP stream was presented for 100ms (25ms rise and fall time) with 20ms between notes. The distractor task included three condition types: no perceptual load (NL), low perceptual load (LL), and high perceptual load (HL). The participant was presented with an RSAP stream and either asked to ignore it (NL), detect infrequent notes of a much higher frequency (two octaves above the frequency range used for non-targets: 1046–2093 Hz) (LL), or detect infrequent tones that were double the duration (200ms) as non-target tones (HL). Previously published dual task studies have utilized similar RSAP streams with frequency and duration changes identifying targets [64–67]. Preliminary data in the lab confirmed that the duration change was more difficult to detect than the frequency/pitch change and was thus assumed to require more attentional resources to detect. Each RSAP stream had a 25% probability of containing no frequency or duration targets, a frequency target only, a duration target only, or both a frequency and duration target resulting in a 50% probability of a target being present for the LL and HL conditions. After each trial, participants were asked to respond first to the CTOJ task then report with a “yes” or “no” button press whether they observed a target for that trial. Each load condition was completed in a separate block, and participants were able to take breaks between blocks. The order that participants completed the load condition blocks was randomized and counterbalanced across participants.

### Data analysis

#### Crossmodal temporal order judgment task

Participants who completed both RSVP and RSAP experiments were included in the analysis with participants who completed one experiment because the experimental effects did not differ in this subgroup. Percent flash first reports were calculated for each SOA within load condition for each participant. Percent flash first reports were then averaged across participants. All statistical analyses were completed using SPSS software. We conducted a Repeated Measures Analysis of Variance (RMANOVA) on percent flash first reports with SOA and perceptual load as within-subjects factors separately for the RSVP and RSAP experiments. We also calculated the partial η^2^ for the perceptual load main effect, SOA main effect, and the SOA by load interaction to determine whether auditory and visual distractors had similar effect sizes on CTOJ performance. The effect size was calculated post-data collection and was not used to determine sample size for the experiment. We then conducted paired sample t-tests between NL and LL/HL to compare differences in percent flash first reports across perceptual loads for each SOA. Alpha error was controlled by adjusting the alpha level to p = .0017 (.05/30 comparisons). To compare across the RSVP and RSAP experiments, we calculated difference scores (HL-NL and LL-NL) in accuracy for each SOA excluding 0ms since there is no correct answer. We then conducted a RMANOVA on the difference scores with SOA, sign (positive versus negative SOA), and perceptual load as within-subjects factors and distractor modality as a between-subjects factor because few participants completed both experiments. Significant effects were explored using post-hoc paired sample t-tests and a bonferroni-adjusted alpha level of p = .0021 (.05/24 comparisons).

#### Calculation of the psychometric function

We individually fit each participant’s percent flash first reports across SOA data to a psychometric function using the curve fitting toolbox in Matlab for each perceptual load using the following four factor sigmoidal function [68,69]:

$$y=\left(A-D\right)/\left(1+\left({\left(\frac{x}{C}\right)}^{B}\right)\right)+D$$

We used the following starting values for each of the four factors: A (upper asymptote) = 100, B (slope) = 5, C (inflection point) = 0, D (lower asymptote) = 0. Furthermore, A was restricted to a range of 75–100, and D was restricted to a range of 0–25. Participants were excluded from this component of the data analysis if the r^2^ value of their psychometric function was less than 75% for any perceptual load. We then determined the point of subjective simultaneity (PSS) as the inflection point (factor C in the above equation) which indicates the point on the curve for which participants are equally likely to report that the flash or beep occurred first [11]. We calculated the negative just noticeable difference (nJND) as the difference in SOA between 25% and 50% flash first reports and the positive JND (pJND) as the difference in SOA between 50% and 75% flash first reports. We conducted RMANOVAs on the PSS, nJND, and pJND values separately with load as a within-subjects factor and distractor modality as a between-subjects factor. We then conducted paired-sample t-tests for the PSS, nJND, and pJND between NL and LL/HL separately for the visual and auditory distractor versions of the task. Alpha error was controlled by adjusting the alpha level to p = .0125 (.05/4 comparisons). We determined the effect size of the influence of load on the positive and negative JNDs by calculating the Cohen’s d for the NL/HL difference scores for both auditory and visual distractors to determine whether the effect sizes were equivalent across distractor modalities.

#### Performance on the distractor task

We calculated percent accuracy on the distractor task for each participant across SOAs separately for each load and distractor modality. We then conducted a RMANOVA on accuracy with perceptual load as a within-subjects factor and distractor modality as a between-subjects factor.

## Results

Participants completed a dual task paradigm that included a CTOJ task and a distractor task that was composed of either visual or auditory distractors and varied in perceptual load (NL, LL, HL). These tasks were used to determine whether directing attention away from the CTOJ task would decrease crossmodal temporal acuity and whether the modality of the distractor modulated this effect. Participants judged the relative order of a visual flash and auditory beep separated by varying SOAs and reported which they perceived as coming first. Average percent visual first reports were calculated for each SOA and load condition separately for the visual and auditory distractors.

### Performance on the crossmodal temporal order judgment task

**Fig 2:**
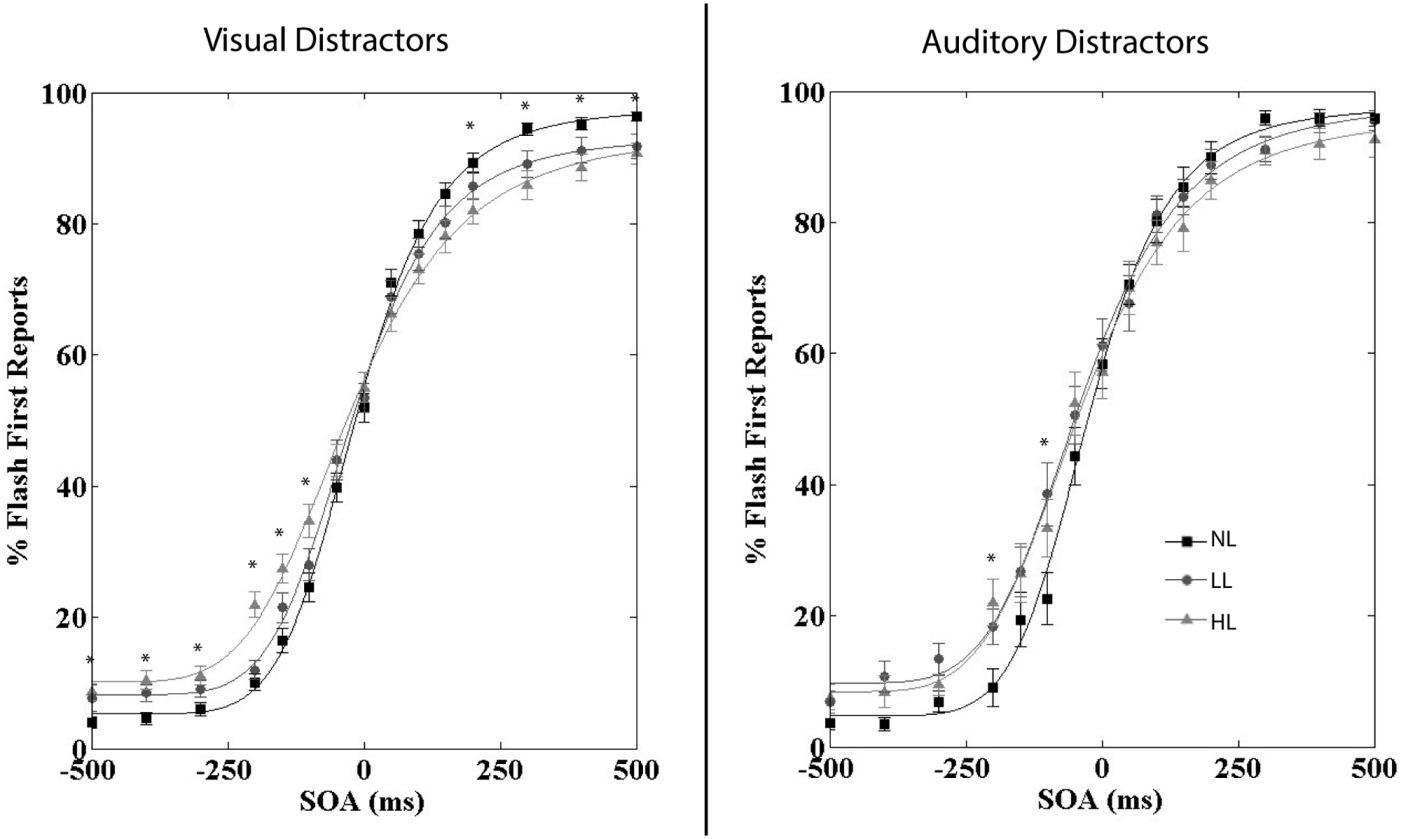
Percent flash first reports across SOA for the CTOJ task separated by visual versus auditory distractor tasks. SOA significantly influenced the percent of flash-first reports with positive SOAs (visual leading) resulting in more visual first reports. SOA and perceptual load significantly interacted for both distractor modalities indicating that perceptual load modulates performance on the CTOJ task. Error bars represent the SEM. * indicate significant differences between NL and HL and/or NL and LL at the Bonferroni-corrected alpha level of p < .0018.

We conducted a RMANOVA on percent flash reports for the visual distractor version of the task with perceptual load and SOA as within-subjects factors. We found a significant main effect of SOA [F(14,1008) = 583.44, p < .001; partial η^2^ = .890], indicating that our CTOJ task was successful in testing crossmodal temporal performance. (Fig 2) Perceptual load did not significantly influence percent flash reports [F(2,144) = 0.44, p = .643; partial η^2^ = .006]; however, the SOA by perceptual load interaction was significant [F(28,2016) = 8.30, p < .001; partial η^2^ = .103], indicating that perceptual load did alter percent flash first reports differently across SOAs. We next conducted paired-sample t-tests between loads at each SOA. The following SOAs were significant after correcting for multiple comparisons: NL/LL [no SOAs] and NL/HL [-500 (t(72) = 3.37, p = .001); -400 (t(72) = 4.11, p = 1.04x10^-4^); -300 (t(72) = 4.11, p = 1.04x10^-4^); -200 (t(72) = 7.15, p<10^−5^); -150 (t(72) = 5.28, p<10^−5^); -100 (t(72) = 4.09, p = 1.11x10^-4^); 200 (t(72) = 4.00, p = 1.52x10^-4^); 300 (t(72) = 4.08, p = 1.15x10^-4^); 400 (t(72) = 3.43, p = .001); 500 (t(72) = 3.57, p = .001)].

We conducted a RMANOVA on percent flash reports for the auditory distractor version of the task with perceptual load and SOA as within-subjects factors. We found a significant main effect of SOA [F(14,392) = 273.29, p < .001; partial η^2^ = .907], indicating that our CTOJ task was successful in testing crossmodal temporal performance. (Fig 2) The main effect of perceptual load approached significance [F(2,56) = 3.12, p = .052; partial η^2^ = .100]; however, the SOA by perceptual load interaction was significant [F(28,784) = 3.79, p < .001; partial η^2^ = .119], indicating that perceptual load did alter percent flash reports more strongly at particular SOAs. We next conducted paired-sample t-tests between loads at each SOA. The following SOAs were significant after correcting for multiple comparisons: NL/LL [-100 (t(28) = 3.85, p = .001)] and NL/HL [-200 (t(28) = 4.32, p = 1.77x10^-^4)]. Taken together, our results clearly demonstrate that increasing perceptual load in both the visual and auditory modalities interferes with performance on the CTOJ task.

### Comparisons of crossmodal temporal order judgment performance across distractor modalities

Because both visual and auditory distractors disrupted CTOJ performance, difference scores (HL-NL or LL-NL) in percent accuracy were calculated for both the visual and auditory distractor versions of the CTOJ task to compare across distractor modality. We conducted a RMANOVA on the difference scores with perceptual load, SOA, and sign (positive versus negative SOAs) as within-subjects factors and distractor modality as a between-subjects factor. The main effect of load was significant [F(1,100) = 5.337, p = .023] indicating that difference scores were larger overall for HL (difference of 6.2) than LL (difference of 4.2). The main effect of distractor modality was not significant [F(1,100) = .040, p = .841] indicating that visual and auditory distractors lead to similar effects on CTOJ performance. The interaction between SOA and sign [F(6,600) = 2.839, p = .010] was significant, indicating that difference scores were larger for auditory-leading trials (mean difference of 6.6 for auditory-leading and 3.8 for visual-leading) but only at particular SOAs. However, post-hoc comparisons between positive and negative SOAs were not significant for visual or auditory distractors at the Bonferroni-corrected alpha level of p = .0021. The interaction between SOA and load [F(6,600) = 2.181, p = .043] was also significant, indicating that the effect of load on difference scores depended on the SOA. However, post-hoc comparisons between HL and LL difference scores were only significant for the -200ms SOA for visual distractors once correcting for multiple comparisons [t(72) = 6.59, p<10^−^5]. Taken together, these results indicate that the strongest modulators of difference scores were the perceptual load of the distractors and the SOA of the CTOJ stimuli and that the modality of the distractors did not have a significant influence.

**Fig 3:**
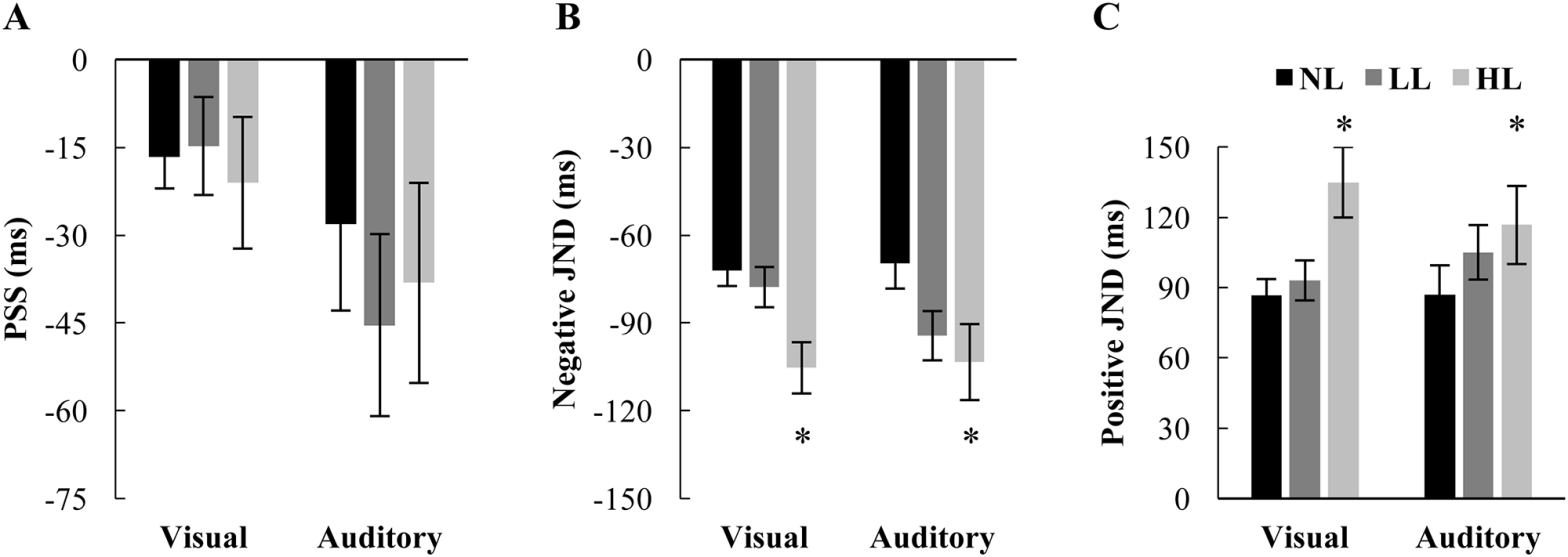
Features of the psychometric function. Individual participant data was fit with a psychometric function for each perceptual load. The resulting mean PSS (A), nJND (B), and pJND (C) are shown grouped by the modality of the distractor task. Both the nJND and pJND, but not the PSS, increased with increasing load. No significant effects of distractor modality were found. Error bars represent SEM. * Indicate significant differences (p < .0125) as compared to NL.

Average visual-first reports for each SOA were fit to a sigmoid curve for each participant separately for each load. The PSS (representing the inflection point of the sigmoid) and positive and negative JNDs (representing temporal acuity) were calculated for each load and participant. (Fig 3) A RMANOVA of the PSS with load and modality as factors revealed no significant main effects, indicating that the PSS did not change across load [F(2,186) = 0.83, p = .439] or distractor modality [F(1,93) = 1.07,p = .304], nor did they interact [F(2,186) = 0.46,p = .631]. (Fig 3A) Perceptual load did significantly influence both the negative (Fig 3B) [F(2,170) = 17.37, p < .001] and positive (Fig 3C) [F(2,166) = 12.65, p < .001] JNDs. Neither the distractor modality nor the interaction between modality and load were significant for positive [main effect of modality: F(1,83) = 0.08,p = .780; interaction: F(2,166) = 1.09,p = .340] or negative [main effect of modality: F(1,85) = 0.04,p = .834; interaction: F(2,170) = 1.751,p = .177] JNDs. Taken together, this indicates that while increasing perceptual load led to decreased crossmodal temporal acuity, the modality of the distractor did not influence this effect. Paired samples t-tests demonstrate that the negative JND for the visual distractor version of the task [NL: -72.1, LL: -77.8, HL: -105.4] was significantly larger between NL/HL [t(59) = 4.82,p < .001; Cohen’s d = .62] when correcting for multiple comparisons but not between NL/LL [t(59) = 1.05, p = .296]. On the auditory distractor version of the task [NL: -69.7, LL: -94.4, HL: -103.4], HL was significantly larger as compared to NL [t(26) = 3.12,p = .004; Cohen’s d = .41] when correcting for multiple comparisons but not between NL/LL [t(26) = 2.44, .021]. Positive JNDs significantly increased between the NL and HL conditions but not between the NL and LL conditions for the visual and auditory distractor versions of the task [Visual Means: NL: 86.6, LL: 93.1, HL: 134.9] [Visual: NL/LL: t(59) = 1.11,p = .296; NL/HL: t(59) = 3.94,p < .001; Cohen’s d = .48] and [Auditory Means: NL: 86.9, LL: 105.0, HL: 116.7] [Auditory: NL/LL: t(26) = 2.14,p = .042; NL/HL: t(26) = 2.92,p = .007; Cohen’s d = .33].

### Distractor task performance (Fig 4)

**Fig 4:**
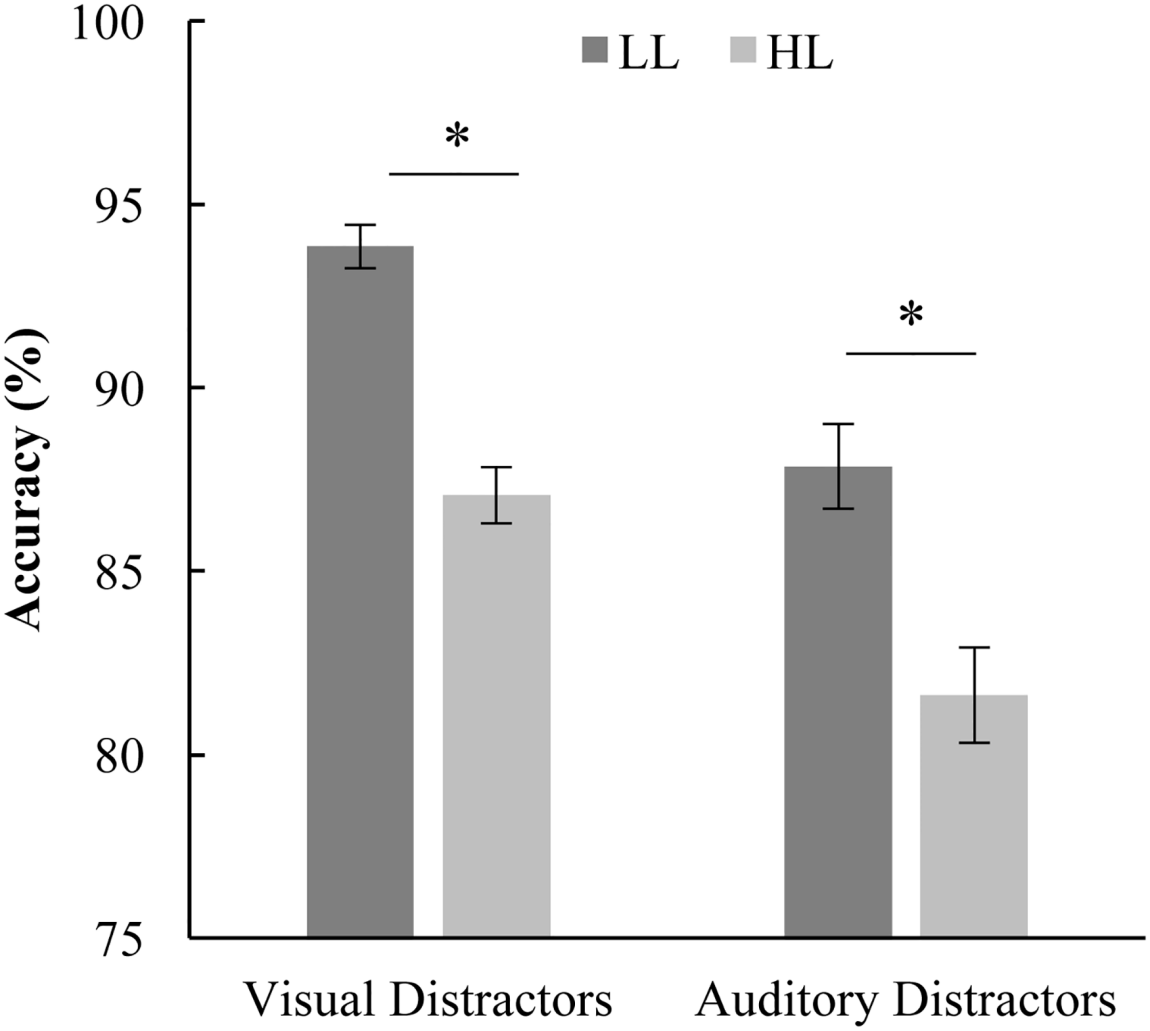
Performance on the visual and auditory distractor tasks. Accuracy was lower for HL compared to LL for both visual and auditory distractors. Additionally, accuracy was higher for the visual distractor task then the auditory distractor task. Error bars represent SEM. * Indicate significance differences between LL and HL.

Concurrent with the CTOJ task, participants viewed a rapid serial visual presentation (RSVP) or a rapid serial auditory presentation (RSAP). Targets were present in 50% of the trials. We conducted a RMANOVA with response accuracy on the distractor tasks as the dependent variable and perceptual load (LL or HL) as a within-subjects factor and modality of the distractor task as a between-subjects factor. Response accuracy was significantly influenced by perceptual load [F(1,98) = 66.74, p < .001] and was higher for LL than HL for both distractor modalities [overall mean accuracy of 95.83 for LL and 88.43 for HL], indicating that the high load versions of the distractor task were more difficult. (Fig 4) This suggests that the HL versions of the distractor tasks draw more attention from the CTOJ task. The modality of the distractors also significantly influenced response accuracy [F(1,98) = 29.21, p < .001] with the visual distractors leading to greater accuracy as compared to auditory distractors [overall mean accuracy of 94.33 for visual distractors and 89.93 for auditory distractors], indicating that the auditory distractor task was more difficult than the visual distractor task. The interaction between distractor modality and load was not significant [F(1,98) = 0.12, p = .734].

## Discussion

### Conclusions

The present study investigated the interactions between attention and multisensory temporal processing by utilizing a dual-task paradigm to reduce the attentional capacity available to process multisensory temporal information. Participants completed a CTOJ task for which they were asked to judge the temporal order of a flash and beep presented at various SOAs while also directing their attention to a secondary distractor task for which they detected a target stimulus within a stream of visual or auditory distractors. We also tested whether the modality of the distractor task would differentially affect performance on the primary CTOJ task. We found decreases in performance on the CTOJ task with increasing visual and auditory perceptual load. Specifically, we found a significant SOA by load interaction in the RMANOVA for visual-first reports and a significant main effect of load in the RMANOVA for accuracy difference scores. Additionally, both the negative and positive JND increased with increasing visual and auditory load. Taken together, these results strongly suggest that attention promotes greater crossmodal temporal acuity and that reducing the attentional capacity available to process multisensory stimuli is detrimental to multisensory temporal processing.

Interestingly, the effect of the distractor task was not uniform across SOAs as evidenced by significant interactions between SOA, sign, and load on difference scores. Participants maintained a relatively high accuracy at the longest SOAs, suggesting that the distractor task did not simply affect the overall performance level. We also found that participants’ performance was more strongly affected for negative (auditory-leading) SOAs. Because of the relative differences in the speed of light versus sound, light from an audiovisual event will often reach our eyes before the corresponding sound reaches our ears. Thus, the most commonly encountered SOAs in natural environments are visual leading. Given the greater detriments to crossmodal temporal performance for auditory-leading SOAs, our results indicate that this less encountered temporal relationship may rely more heavily on attentional resources to be discernable.

Although our study provides strong evidence that attention promotes more accurate crossmodal temporal processing, the observed interaction between multisensory temporal processing and attention may differ depending on the experimental manipulation of attention. For example, temporal but not spatial crowding appears to disrupt crossmodal temporal acuity [53,54]. Additionally, attention may interact differently with multisensory temporal processing when unisensory stimuli are to be integrated (temporal window of integration) [55] versus when the temporal relationship between unisensory stimuli is being actively compared (crossmodal temporal acuity). Thus, a reduced attentional capacity may have a different impact on the temporal window of integration than what is predicted by the current study findings. Unfortunately, investigating the effects of perceptual load on the temporal window of integration is problematic given that increased perceptual load has been linked to decreases in multisensory integration [38]. Future studies will also need to investigate whether increased perceptual load equally disrupts crossmodal temporal acuity for higher order multisensory stimuli since attention may interact differently with multisensory temporal processing depending on task-specific features [55] and because complex stimuli have larger temporal windows of integration [70].

Contrary to our hypothesis, we found no differences between the auditory and visual distractor tasks in their effects on crossmodal temporal acuity. Both distractor tasks resulted in poorer CTOJ performance and increases in the JND that were statistically indistinguishable across modalities. We also did not observe changes in the PSS with increasing load when the CTOJ task was accompanied by either the visual or auditory distractor task. Notably, our results stand in contrast to the theory of prior entry, which predicts that modality-specific attention should alter the speed of neural processing of stimuli in the corresponding modality. For example, if the visual distractor task reduced the capacity to process visual stimuli, participants should show greater accuracy for auditory-leading and poorer accuracy for visual-leading pairs of stimuli. Prior entry has been established in selective attention paradigms and may function differently in dual task paradigms. Alternatively, it is possible that in our dual task paradigm, the visual and auditory distractor tasks reduce capacity in a supramodal rather than modality-specific manner. Previous studies have investigated whether attentional capacity is supramodal or modality-specific (for a general discussion see [67]). However, these studies have generated conflicting results with some studies finding that attentional capacity is independent across sensory modalities [64] and others finding that perceptual load in one modality interferes with performance and neural processing in a different modality [71,72].

We did find differences in performance on the distractor task as a function of perceptual load and distractor modality. For both the auditory and visual distractors, we found decreases in accuracy with increasing perceptual load. This indicates that for both modalities, the high load feature that participants were instructed to detect (numbers for the RSVP and longer duration for the RSAP) was more difficult and thus likely demanded more attentional resources. We also found that the auditory distractor task resulted in lower accuracy than the visual distractor task and far more participant exclusions due to poor performance. This suggests that the visual and auditory distractor tasks may have demanded unequal attentional resources. Additionally, this study had approximately twice the number of participants in the RSVP versus RSAP experiments. These differences between the two versions of the task may have acted as a confound and masked some relevant differences between the effects of distractor modality. However, these differences across the visual and auditory distractor tasks are unlikely to be major confounds because of the almost indistinguishable effects of increasing load on CTOJ performance across the two distractor modalities. Additionally, measures of effect size for load and SOA were similar across distractor modalities, suggesting that increasing visual versus auditory load had similar effects on CTOJ performance. However, future studies utilizing visual and auditory distractor tasks that are more equivalent in their difficulty and sample sizes are needed to confirm whether auditory and visual distractors equally effect crossmodal temporal acuity. We also noted a slightly unequal gender ratio in our included participants and a very unequal gender ratio in our excluded participants. Very little is known about the potential influence of gender on multisensory integration; thus, future research may be needed to evaluate potential gender differences in the effects of attention on multisensory processing.

### Potential neural mechanisms

Much is known about how the brain represents the temporal relationships between unisensory events. This knowledge can be useful to help frame our understanding of how attention alters multisensory temporal processing. One such framework is the temporal window of integration (TWIN) model that was proposed by Colonius and Deiderich [10,73]. In this model, unisensory information is initially processed independently and is thought to be engaged in a “race.” The next stage of the model, the integration stage, includes all processes after the initial unisensory “race.” If multiple unisensory signals enter the integration stage within the same window of time, they will be integrated into a multisensory percept [73]. In this framework, a reduced attentional capacity could alter the initial processing of unisensory signals such that the initial signal is delayed and initiates the integration stage abnormally late, thus increasing the interval for which the second signal could reach the integration stage. Additionally, attention could alter the length of the integration stage such that reducing the attentional capacity for either modality elongates the length of the integration stage for signals from either modality. Our data more strongly support attention acting at the integration stage because the auditory and visual distractor tasks had equivalent effects on CTOJ performance.

Although the TWIN model is helpful in our understanding of the neural mechanisms of the effects of attention on multisensory temporal processing, it may not apply in the case of crossmodal temporal acuity when participants are actively contrasting (as opposed to integrating) multisensory temporal information. Thus, instead of unisensory signals needing to arrive within a set time-period of each other, the temporal pattern of these signals may be compared in multimodal brain areas or across unimodal areas. For example, the superior temporal sulcus (STS) has been demonstrated to differ in its activity depending on the temporal structure of multisensory events [74–77]. Areas such as the STS may compare the relative onsets or temporal profiles of each unisensory signal arriving from their respective sensory cortices to determine their temporal relationship. A reduced attentional capacity could delay the onset of unisensory information reaching STS or enlarge the time over which unisensory information feeds into STS. Any of these potential mechanisms would lead to a decreased ability to discern temporal order. Additionally, alterations in synchronous oscillatory activity between unisensory and multisensory areas may be the underlying mechanism for the effects of attention on crossmodal temporal acuity. Synchronous coupling of ongoing oscillations across unisensory and multisensory areas has been shown to be important for multisensory integration [78], and audiovisual synchrony has been demonstrated to influence oscillations at gamma frequencies [79]. The relative contributions of all the aforementioned potential neural mechanisms could be assessed using electroencephalography (EEG) and comparing changes in neural activity with increasing load to the corresponding changes in performance on the CTOJ task. Specifically, changes in oscillatory amplitude, phase locking, and coherence either from trial to trial or between groups of electrodes could be used to assess the role of synchrony and power in ongoing oscillatory activity. Additionally, changes in behavior with increasing load could be linked to changes in peak amplitude, width, and latency to assess the role of changes in the onset or temporal signature of unisensory signals.

### Implications for developmental disorders

In characterizing the relationship between attention and temporal multisensory processing, the present study may shed light on the multisensory deficits present in many neurodevelopmental disorders. Enlargements in the temporal window of integration have been found for autism spectrum disorders (ASD), dyslexia, and schizophrenia [6,16,17,19]. These disorders also show alterations in the control of top-down attentional functions [80–82]. Although the present study investigates the effect of attention on crossmodal temporal acuity in typically developed adults, our findings raise important questions as to the cause of the enlarged temporal window in developmental disorders. The enlarged temporal window could result from an alteration in sensory functioning and/or differences in top-down attention. Future studies comparing differences in performance between neurotypicals and those with developmental disorders on this CTOJ task with increasing load may cast light upon this question. Additionally, future studies could compare individual differences in attentional capacity with measures of the temporal window of integration or crossmodal temporal acuity to determine whether participants with relatively limited attention resources are likely to have greater difficulty distinguishing temporal information across modalities.

Knowing whether changes in sensory versus attentional processes have a stronger impact on multisensory temporal processing could help in the advancement of potential remediation strategies for developmental disorders. For example, differences in the temporal window of integration have been linked to speech deficits in ASD [83] and the relative timing between auditory and visual signals influences the effects of visual speech on auditory speech perception [84]. Additionally, the temporal window can be narrowed through perceptual training [13,14,77]; however, it has currently not been demonstrated whether narrowing the temporal window leads to improved speech perception in ASD. Training individuals with developmental disorders in the realm of attentional control to help improve attentional capacity either alone or in conjunction with temporal perceptual training may lead to greater improvements in speech perception. Overall, the results of this study add to our understanding of how attention interacts with multisensory integration. Importantly, we have provided a clear link between attentional capacity of both the visual and auditory modalities and a person’s ability to discriminate small temporal differences. The findings of this study have important implications not only for our understanding of developmental disorders but also for the design of multisensory warning signals and other multisensory stimuli for entertainment purposes that are increasingly being incorporated into our technology [85].
